# Pilot study comparing simulation-based and didactic lecture-based critical care teaching for final-year medical students

**DOI:** 10.1186/s12871-015-0109-6

**Published:** 2015-10-21

**Authors:** Orsolya Solymos, Patrick O’Kelly, Criona M. Walshe

**Affiliations:** 1 Department of Anaesthesia and Critical Care Medicine, Royal College of Surgeons in Ireland, Smurfit Building, Beaumont Hospital, Dublin, Ireland; 2 Department of Nephrology, Beaumont Hospital, Beaumont Road, Dublin 9, Ireland

## Abstract

**Background:**

Simulation-based medical education has rapidly evolved over the past two decades, despite this, there are few published reports of its use in critical care teaching. We hypothesised that simulation-based teaching of a critical care topic to final-year medical students is superior to lecture-based teaching.

**Methods:**

Thirty-nine final-year medical students were randomly assigned to either simulation-based or lecture-based teaching in the chosen critical care topic. The study was conducted over a 6-week period. Efficacy of each teaching method was compared through use of multiple choice questionnaires (MCQ) - baseline, post-teaching and 2 week follow-up. Student satisfaction was evaluated by means of a questionnaire. Feasibility and resource requirements were documented by teachers.

**Results:**

Eighteen students were randomised to simulation-based, and 21 to lecture-based teaching. There were no differences in age and gender between groups (*p* > 0.05).

Simulation proved more resource intensive requiring specialised equipment, two instructors, and increased duration of teaching sessions (126.7 min (SD = 4.71) vs 68.3 min (SD = 2.36)).

Students ranked simulation-based teaching higher with regard to enjoyment (*p* = 0.0044), interest (*p* = 0.0068), relevance to taught subject (*p* = 0.0313), ease of understanding (*p* = 0.0476) and accessibility to posing questions (*p* = 0.001).

Both groups demonstrated improvement in post-teaching MCQ from baseline (*p* = 0.0002), with greater improvement seen among the simulation group (*p* = 0.0387), however, baseline scores were higher among the lecture group. The results of the 2-week follow-up MCQ and post-teaching MCQ were not statistically significant when each modality were compared.

**Discussion:**

Simulation was perceived as more enjoyable by students. Although there was a greater improvement in post-teaching MCQ among the simulator group, baseline scores were higher among lecture group which limits interpretation of efficacy. Simulation is more resource intensive, as demonstrated by increased duration and personnel required, and this may have affected our results.

**Conclusions:**

The current pilot may be of use in informing future studies in this area.

## Background

Critical care medicine poses challenges for the education of both medical students and junior doctors. There exists an inherent difficulty in imparting knowledge on complex topics that are difficult to grasp for medical students who often have had minimal exposure to direct patient care. This is particularly true within the critical care environment, the nature of which leads to limited opportunities for bedside teaching given dangers of exposing this vulnerable patient subpopulation to large numbers of students. Thus, critical care teaching frequently relies on provision of tutorial and didactic lecture sessions.

Simulation involves the creation of an artificial depiction of complex clinical situations which are infrequently encountered. If the fidelity of the simulator is sufficient, then learning is facilitated through immersion, reflection and feedback but carries the benefit of protection of both patient and learner from risks associated with these clinical situations [1]. Simulation-based medical education is gaining popularity with much discussion in the literature of its proposed merits [2]. Critical care teaching may be particularly suited to simulation-based education as it is often not feasible to demonstrate live critical care scenarios to medical students in the hospital setting. A completely safe version mimicking such complex scenarios can easily be acted out in the Simulation Laboratory. And yet our literature research has revealed few studies of its use in the education of medical students in critical care.

The installation of two Laerdal SimMan 3G mannequins by the Royal College of Surgeons in Ireland (RCSI) for the training purposes of medical students has facilitated simulation sessions to be delivered in the medical discipline, the surgical discipline and in basic and advanced cardiac life support.

The aim of this study is to examine whether simulation-based teaching of a critical care topic to final-year medical students is superior to lecture-based teaching.

## Methods

Ethics approval was obtained from the RCSI Research Ethics Committee (REC). The study population comprised final-year medical students rotating through a 1-week Anaesthesia and Critical Care attachment as part of their final-year medicine curriculum within the RCSI.

### Participants and setting

Students are assigned to the Department of Anaesthesia in groups of five to eight students per week. Following informed consent, each group was randomised to one of two teaching methods, simulation-based teaching or didactic lecture. The chosen topic was recognition of the critically ill patient at ward level with a focus on sepsis. Each method of delivery of this teaching session was carefully prepared to deliver the same key concepts to allow the student to recognise the deteriorating patient at ward level, diagnose and manage sepsis, as per the Surviving Sepsis Guidelines [3]. All teaching was delivered by the same RCSI Clinical Lecturer, and Consultant in Anaesthesia and Critical Care.

### Measurements

#### Questionnaires and MCQ tests

We compared the efficacy of the two teaching methods through the use of multiple choice questionnaire papers (MCQ). Each MCQ comprised five questions with five true or false stems, and results were marked out of a maximum of 25. Prior to the teaching session each student completed the baseline multiple choice questionnaire (baseline MCQ). Students completed a second MCQ immediately following the teaching session (post teaching MCQ).

To ensure that the participants were not at a disadvantage or that they did not perceive a disadvantage by participation in this study, all students were offered the opportunity to return to avail of the teaching delivered by the teaching method to which they were not randomised. This session was held 2 weeks following the initial teaching session. Prior to this supplementary teaching session they were asked to complete the third and final MCQ (2-week follow up MCQ). The results of all MCQ examinations were fed back to each participating student upon completion of this final teaching session and MCQ examination.

#### Resource requirements

The resource requirements for each teaching method were assessed by looking at the time required for didactic lecture preparation and delivery versus preparation and delivery of simulation scenarios, in addition to personnel and equipment resources required for each mode of teaching.

We utilised a high fidelity, event driven simulator. This required two instructors, one to engage within the scenario with the students, to protect against patient demise in the event of poor student performance, and a second to coordinate computer driven physiological responses dependent on intervention implemented by students within the scenario.

#### Qualitative analysis

Finally, qualitative assessment was analysed by means of a questionnaire filled out by the students following each teaching session. These were designed to assess clarity of teaching, student enjoyment, accessibility of learner to pose questions etc. to gain student perceptions of the teaching style.

### Statistical analysis

Two-sample Wilcoxon rank-sum (Mann–Whitney) test was used to evaluate the results of the student satisfaction questionnaires and to compare the difference in results of the pre- and post-teaching multiple choice questionnaires between the two groups.

Wilcoxon signed-rank test was used to compare the results of the multiple choice questionnaires within the two groups.

Pearson Chi-squared test was used to compare the difference in gender composition between the two groups.

## Results

Forty-one final year medical students comprised the study population. Two students were excluded from the study due to non completion of the pre- or the post-teaching MCQ.

Median ages (interquartile range IQR) were 24 (24–25) in the simulation group and 24 (23–27) in lecture-based teaching group (*p* = 0.7). Sex distribution in the study population was not statistically significant, 43.6 % female (17/39), 56.4 % male (22/39), (*p* = 0.1).

All students included in study population completed baseline and post teaching MCQs. Sixteen students returned to avail of the learning method to which they were not randomised and completed the 2-week follow up MCQ; 5 of the simulation-based teaching group (28 % (5/18)) and 11 of the lecture-based teaching group (52 % (11/21)). Results of all MCQ papers are depicted in Table 1. There was a significant difference in the improvement from baseline and post teaching MCQ in the simulation group compared to lecture 6.8 (21.1 - 14.3) vs. 4.5 (21.5 - 17), *p* = 0.0387. The results of 2-week follow up MCQ were lower in both groups than post teaching results. Although this margin was smaller in the simulation group 1.3 (19.8-21.1) vs. 3.6 (17.9 - 21.5), this was not statistically significant (*p* = 0.167).

**Table 1:** Results of MCQ at baseline, post teaching and at 2 week follow up. Scores are marked out of a total of 25, results are depicted as mean +/− standard deviation for each group

Method	Baseline MCQ Mean(+/−SD)	Post-teaching MCQ Mean(+/−SD)	Two-week follow up MCQ Mean(+/−SD)
Simulation	14.3(2.2)	21.1(1.8)	19.8(3)
Lecture	17(3)	21.5(3.1)	17.9(2.5)

Duration of preparation and delivery of each teaching session are outlined in Table 2. In terms of financial outlay, the SimMan simulator cost Euro 60,000. There was no financial implication for the provision of the didactic lecture as all necessary equipment was made freely available by RCSI and Beaumont Hospital.

**Table 2:** Duration of preparation and delivery of each mode of teaching

Method of teaching	Mean duration of teaching session	Duration of preparation
Simulation	126.7 min	Training of teachers on simulator: 240 min
	(SD = 4.71)	Scenario Preparation: 180 min
		Simulator Start-up: 10 min
Lecture	68.3 min	Lecture Preparation: 180 min
	(SD = 2.36)	Laptop/projector Start-up: 3 min

*SD* standard deviation

Tables 3 and 4 depict student perceptions of the teaching experience.

**Table 3:** Summary of student perceptions of the experience of each mode of teaching. Students were asked to rank the teaching experience from 1 – 5 (one strongly disagree; five strongly agree) for each of the following

Method of teaching	Organisation	Amenable to questions	Pace	Duration	Addressed subject matter
Simulation	4.9	4.9	4.5	2.3	4.8
Lecture	4.6	4.6	4.2	2.5	4.3
p value	0.037	0.011	0.703	0.077	0.019

**Table 4:** Summary of student perceptions of quality of each mode of teaching. Students were asked to rank their thoughts on the quality of the teaching out from 1–10 (one being the worst ten the best) strong for each of the following

Method of teaching	Enjoyment	Interest	Relevance	Ease of understanding
Simulation	8.7	9.2	9.7	9.6
Lecture	7.2	8	9	9.2
p value	0.0044	0.0068	0.0313	0.0476

## Discussion

The use of simulators in medical education has vastly increased in recent years [4], and there are now available a wide variety of commercially available products. In this pilot study, we utilised a high fidelity simulator to engage the students in a teaching session which focused on recognising a patient at ward level who is critically ill, with sepsis as the cause of clinical deterioration, and explore the merits and resources required to run simulation-based teaching of a critical care topic to medical students.

Critical care medicine lends itself particularly well to simulation training [5], given the inherent difficulties of teaching large numbers of students on rare and serious clinical events in a vulnerable patient population [6, 7]. Despite this, there are limited studies examining the use of the simulator in critical care. Schroedl et al. examined simulation based education as a means to teach topics pertinent to medical ICU and demonstrated higher scores in skills assessment among simulation training as compared to controls [8]. Others have investigated the use of the simulator to teach medical emergencies, with varied results. Ruesseler et al. studied the use of simulation training in medical emergencies in a group of final year medical students and found performance in OSCE stations superior among simulation students, compared to controls [9]. In a study comparing didactic lecture to use of simulation to teach perioperative ultrasound higher test scores were demonstrated among the simulation group [10]. Daniels et al. demonstrated significantly higher performance in the simulation group among residents and nurses being taught obstetrical emergencies compared to didactic teaching and Hallikainen et al. demonstrated an improved task performance among students taught anaesthesia induction among the simulator group, as compared to those learning by means of observed practice [11]. In a study by Morgan et al., simulation was compared to video based learning to teach a number of medical emergencies, however, the investigators determined no differences in quantitative assessments between each group, although students found the simulator sessions more enjoyable and valuable [12]. Tan et al. demonstrated equal efficacy between screen based simulation and conventional lecture in a group of students taught medical emergency management [13]. However, we could not identify studies examining the use of the simulator for the purposes of critical care education.

In terms of the critical care topic utilised in this study, we chose the deteriorating patient at ward level, because this is a particularly pertinent clinical scenario frequently encountered by doctors in their first year of clinical practice. The focus was sepsis because of the time sensitive nature of interventions that have been shown improve patient outcomes [14]. The use of simulation training has a number of advantages over traditional medical education methods including the provision of a safe environment for both teacher and student during training in risky procedures, unlimited exposure to rare but complicated and important clinical events, the ability to plan and shape training opportunities rather than waiting for a suitable situation to arise clinically, the ability to provide immediate feedback, the opportunity to repeat performance as well as the opportunity for team training. We could find no study in the literature exploring the use of simulation education on this topic, sepsis, to either medical students, or junior doctors.

Simulator based teaching proved to be more resource intensive. In addition to the cost of purchasing the Simulator are the as yet unknown ongoing maintenance costs beyond the 5-year warranty period. In terms of preparation, the simulator required longer time for teachers to be trained in its operation and use, an addition to scenario preparation 420 min vs. 180 min for didactic lecture preparation (Table 2). Similarly, delivery of simulation based teaching proved more resource intensive taking on average twice as long compared to didactic lecture delivery, 126.7 vs. 68.3 min, and necessitating presence of two teachers. Although our results demonstrate a trend toward improved efficacy as demonstrated by greater increase in MCQ scores from baseline to post teaching, there remains the possibility that some of this was due to the greater time spent teaching the topic among the simulation group, for which it is difficult to control, and this may have influenced our results.

In this study we utilised MCQ as a means of assessing the efficacy of education sessions provided, but further study is warranted to explore whether the assessment of skills acquired in carrying out practical tasks such as resuscitation of critically ill patients with sepsis would be more accurately achieved through use of the simulator as a means of assessment. In a study among Radiology trainees [15], Wang et al. demonstrated no improvement in written test scores among groups of radiology trainees receiving didactic lecture versus simulation based training in management of contrast reaction, although performance among simulation based training was superior when tested in a contrast reaction simulation scenario. In the current study, sudents who received simulation-based education demonstrated significantly improved performance in post teaching MCQ from baseline, compared to those who received didactic lecture (Table 1). However, baseline MCQ scores were lower among the simulator group. These results may support the evidence outlined above, that simulation may be more efficacious, although randomisation failure cannot be excluded and further study is warranted.

We compared the sustainability of the acquired knowledge through the completion of an MCQ 2 weeks following the teaching session. This was offered as an entirely optional session, and unfortunately only a small number of students obliged (28 % of simulator group returned for follow up compared to 52 % of the lecture group). We found that both simulation and lecture based groups had lower scores at 2-week follow up compared to post teaching, and although the margin of this deterioration was smaller in the simulation group (1.3 vs. 3.6), this did not achieve significance, (*p* = 0.1670). The non significance of this result may be due, at least in part, to the small numbers of students involved, and there is the possibility of bias given the discrepancy between return rates for each group, and high attrition rate given the small numbers in each group. Other investigators have demonstrated improved knowledge retention among simulation based education compared to didactic lecture, with follow at 2–3 weeks [10]_._ This needs to be further studied.

Students were asked to rate the teaching experience and there was a statistically significant difference in favour of the simulation-based teaching with regard to organisation, and adequate address of chosen subject matter and how amenable the session proved to students in terms of posing questions (Table 3). Students also ranked enjoyment, interest, relevance and ease of understanding of the teacher higher among the simulation teaching (Table 4). That the students enjoyed simulation based education has been commented upon by other investigators, such as the study mentioned above by Morgan et al. [12], and a study by Paskins et al. which evaluated students views on simulation based teaching [16].

The purpose of this pilot was to establish feasibility of the use of the simulator to deliver critical care education to final year medical students, and to establish study population required for further randomised control trial. We acknowledge the limitation of our use of an MCQ as the assessment tool, where Simulation based assessment sessions would likely have proved superior; however resource implications obviated our ability to perform such assessments. Further limitations of this small pilot study include the differences in duration of each teaching session, with the simulation session being almost twice as long in duration and the lower student:teacher ratios which may have influenced our results.

## Conclusions

The need for simulation-based medical education in clinical practice, especially in critical care medicine, is likely to continue or increase given the proposed merits for both students and teachers. Given that simulation based education is both time-consuming and resource intensive, its long-term merits with regard to retaining knowledge and translating into improved patient care need to be further studied and confirmed. While acknowledging the limitations of the current study as outlined above, this study demonstrated increased student enjoyment of simulator based teaching compared to didactic lectures and may be used to inform further studies investigating this important area.

## Abbreviations

MCQ: Multiple choice questionnaire
SD: Standard deviation
RCSI: Royal College of Surgeons in Ireland
